# Calibration and Validation of a PREMIUM-DT Item Bank to Measure the Experience of Drug Therapy for Patients with Severe Mental Illness

**DOI:** 10.3390/jcm11154278

**Published:** 2022-07-22

**Authors:** Sara Fernandes, Laurent Boyer, Xavier Zendjidjian, Anderson Loundou, Jeremie Riedberger, Pierre-Michel Llorca, Pascal Auquier, Guillaume Fond, Collaborators of the French PREMIUM Group

**Affiliations:** 1 CEReSS-Health Service Research and Quality of Life Center (UR3279), Aix-Marseille University, 13005 Marseille, France; laurent.boyer@ap-hm.fr (L.B.); xavier.zendjidjian@ap-hm.fr (X.Z.); anderson.loundou@univ-amu.fr (A.L.); jeremie.riedberger@ap-hm.fr (J.R.); pascal.auquier@univ-amu.fr (P.A.); guillaume.fond@gmail.com (G.F.); 2 Fondation FondaMental, 94000 Créteil, France; pmllorca@chu-clermontferrand.fr

**Keywords:** psychiatry, mental health, schizophrenia, depressive disorders, bipolar disorders, patient-reported experience measures, health services research

## Abstract

The aim of this study was to (1) calibrate an item bank to measure patients’ experience of drug therapy for adult patients with SMIs and (2) develop computerized adaptive testing (CAT) to improve its use in routine practice. This is a cross-sectional, multicentric study involving 541 patients with schizophrenia, bipolar disorder, and major depressive disorder. Analyses based on classical test and item response theories were performed. After 7 highly inter-correlated items and 4 items with low factor loadings were removed, the remaining 26 items were sufficiently unidimensional (RMSEA = 0.069, CFI = 0.969, TLI = 0.963) and showed adequate fit to the generalized partial credit model. There was no differential item functioning by gender, age, care setting, or diagnosis from moderate- to large-magnitude. The mean score was 46.0 ± 16.9 and was significantly higher for patients reporting good medication adherence. The resulting PREMIUM-DT item bank has strong psychometric properties, and CAT facilitates widespread use in clinical settings (an average of 8 items administered, corresponding to a reliability of >0.90). Our results suggest that practical information and information about the side effects of psychotropic treatments and how to cope with them should be targeted as a priority to improve patients’ experience of drug therapy.

## 1. Introduction

Schizophrenia, bipolar disorders, and major depressive disorders are severe mental illnesses (SMIs) that are associated with poor quality of care [1,2,3]. The use of patient-reported experience measures (PREMs) is now recognized as a key measure to improve quality of care [4,5,6]. Most existing questionnaires are in a standardized paper-and-pencil format, which is a barrier to use in routine practice because of the lack of accuracy and workload for patients and clinicians. Computerized adaptive tests (CATs) and item banks based on item response theory (IRTs) can overcome these limitations [7,8,9,10]. As part of the French initiative PREMIUM (Patient-Reported Experience Measure for Improving qUality of care in Mental health), item banks and CATs measuring patients’ reported experience of mental health care are underdeveloped [11]. Drug therapy is a key dimension of the experience of adult patients with SMIs, as identified in our previous work [12]. In all countries of the world, there is a large gap for SMIs, with a mismatch between the need for treatments and their provision and sometimes inadequate treatments [13,14,15]. Poor adherence to medication is also a major challenge in all fields of medicine and is particularly challenging in the psychiatric routine care of SMIs patients [16]. In particular, poor adherence induces impaired prognosis, increased relapse and suicide risk, more frequent and longer hospitalizations, impaired quality of life and professional and social functioning, and excessive costs for society [17,18,19]. New drugs with fewer side effects and long-acting injection forms have been developed to increase adherence with limited effectiveness [20]. Discrepancies in belief systems between the general public and health care professionals may explain this glass ceiling. For example, some patients may believe that drugs could poison them or be addictive. Prescribing drugs should therefore be accompanied by long talk sessions. The concept of shared decision-making has been developed to increase patients’ commitment to their treatment [21,22,23]. In summary, good practice in prescribing drug therapy includes understandable and adapted information and patient commitment to medical decisions.

The aim of this study was to (1) calibrate an item bank to measure the experience of drug therapy for adult patients with SMIs and (2) develop a CAT intended to be in routine practice.

## 2. Methods

### 2.1. Design and Study Setting

In this national, cross-sectional and multicenter study, patients were recruited from January 2016 to December 2021 including inpatient and outpatient departments (i.e., full-time hospitalization, day hospitalization, and outpatient care) of a French teaching hospital (Assistance Publique—Hôpitaux de Marseille, Marseille, France), from the FondaMental Foundation’s expert centers and through an online web survey. All participants gave their informed consent on research participation. This study was approved by the competent ethics committee (CPP-Sud Méditerranée V, Nice, France, 12 November 2014, n°2014-A01152-45).

### 2.2. Study Population

The inclusion criteria were a DSM-5 diagnosis of schizophrenia, bipolar or major depressive disorders [24]; inpatient or outpatient psychiatric care, regardless of current or previous care, duration, or severity of illness; age > 18 years and < 65 years; and being able to read and speak French. The exclusion criteria were mental retardation or decompensated organic illness; vulnerable categories of persons (i.e., pregnant or nursing women, persons under legal protection measures, etc.); inability to fill out a self-administered questionnaire; and withdrawal of consent.

### 2.3. Data Collection

The data collected were as follows:

- Sociodemographic information: sex; age, educational level; marital status; and occupational status.
- Clinical information: diagnosis (schizophrenia, bipolar or major depressive disorders); duration of illness; psychological, social and occupational functioning measured by the Global Assessment of Functioning scale [25] (GAF, ranging from 0 to 100, with a higher score indicating better functioning); medication adherence measured by the Medication Adherence Rating Scale [26] (MARS, ranging from 0 to 10, with higher scores indicating better medication adherence); quality of life (QoL) measured by the medical outcome study 12-item Short Form (SF-12) [27], which describes 8 dimensions: physical functioning (PF), social functioning (SF), role physical (RP), role emotional (RE), mental health (MH), vitality (VT), bodily pain (BP); general health (GH), and two composite scores for physical (PCS) and mental (MCS) quality of life (ranging from 0 to 100, with higher scores indicating better quality of life).
- The drug therapy item bank (PREMIUM-DT) including 37 items, an overall satisfaction item (“Overall, were you satisfied with your drug therapy?”) and a visual analogue scale (VAS) (ranging from 0 to 10). All items were scored on a 5-point Likert scale (“strongly disagree”, “disagree”, “neither agree nor disagree”, “agree”, “strongly agree”) with a “not applicable” response option. The coding of negatively worded items was reversed so that higher scores indicated a greater patients’ experience of drug therapy. The assessment period referred to the four weeks prior to administration.

### 2.4. Statistical Analysis

The PREMIUM project has been described previously, including the statistical analysis plan [11], in divided into four steps: (1) conceptual work and definition of domain mapping; (2) item selection; (3) item bank calibration and CAT simulations; and (4) CAT validation. In this study, only the third step was reported for the PREMIUM-DT item bank. 

#### 2.4.1. Descriptive Analysis

The 37 items of the PREMIUM-DT bank were described and excluded in case of (1) missing value rates >70%; (2) extreme skewness (>95% of response rate in one category or an absolute coefficient >4); or (3) inter-item correlation coefficients > 0.70 were excluded. Internal consistency was evaluated by calculating Cronbach’s alpha coefficient, with α > 0.70 considered to be acceptable [28].

#### 2.4.2. Evaluation of the Assumptions of the IRT Model

The 3 key assumptions of the IRT framework were evaluated: (i) unidimensionality, (ii) local independence, and (iii) monotonicity [7,29,30,31,32,33,34,35,36,37,38]. All the details are presented in Supplementary Materials.

#### 2.4.3. Calibration and Fitting of an IRT Model to the Data

The generalized partial credit model (GPCM) [39] was used to calibrate the responses to the items as in our previous works [40]. The likelihood ratio test [41] as well as Akaike information criterion (AIC) [37] and the Bayes information criterion (BIC) [38] were calculated and compared between the GPCM and the partial credit model (PCM, in which the discrimination parameter is equal across all items) [42] to select the IRT model that best fit the data. The item parameters (discrimination and thresholds) were then estimated under the selected model using the maximum marginal likelihood estimation (MMLE) implemented via the expectation–maximization (EM) algorithm [43]. Items with a discrimination parameter below 0.50 were also considered problematic [44,45], as they were not sufficiently informative and were thus removed from the item bank. The goodness-of-fit was evaluated by computing the infit mean square (Infit MnSq) statistic [46] with an expected value in the range [0.7–1.3] [47].

#### 2.4.4. Evaluation of Differential Item Functioning (DIF)

DIF analyses were performed to see if all items behave in the same way [48,49] according to sex (men vs. women), age (median split: patients 36 years or younger vs. patients older than 36 years), care setting (outpatient vs. inpatient), and psychiatric diagnosis (schizophrenia vs. bipolar disorders vs. major depressive disorders). If an overall DIF was detected at a level of *p* < 0.01, the magnitude was assessed according to Zumbo’s DIF classification by computing the pseudo R^2^ change (ΔR^2^): negligible if ΔR^2^ < 0.13, moderate if 0.13 < ΔR^2^ < 0.26, and large if ΔR^2^ > 0.26 [50]. Items with a large DIF were excluded from the item bank.

Latent trait scores (θ) for each respondent were estimated by Bayesian expected a posteriori (EAP) estimation [51]. Then, a linear transformation was performed to have θ scores ranging from 0 to 100 (the higher the score was, the better the patients’ experience of drug therapy). Item and test information were calculated.

#### 2.4.5. External Validation of the Item Bank

Tests of external validity were based on the following a priori hypotheses: higher levels of patients’ experience of drug therapy (PREMIUM-DT scores) would be associated with higher levels of medication adherence measured by MARS, quality of life measured by SF-12 dimension scores, global functioning measured by GAF, and satisfaction with drug therapy. Discriminant validity was assessed by comparing the mean scores of the PREMIUM-DT item bank according to sociodemographic (i.e., age, sex, educational level, marital status and employment status) and clinical (i.e., care setting, duration of illness and diagnosis) data using Student’s t-test, analysis of variance (ANOVA), and Pearson’s correlation coefficient. The normality of the data was checked using the Q-Q plot.

#### 2.4.6. Elaboration of Item Administration Algorithm

CAT simulations were performed using both real response data (i.e., complete response patterns to items in the final PREMIUM-DT item bank) and simulated data (i.e., after imputation of plausible missing responses using IRT-based estimation).

**Figure 1:**
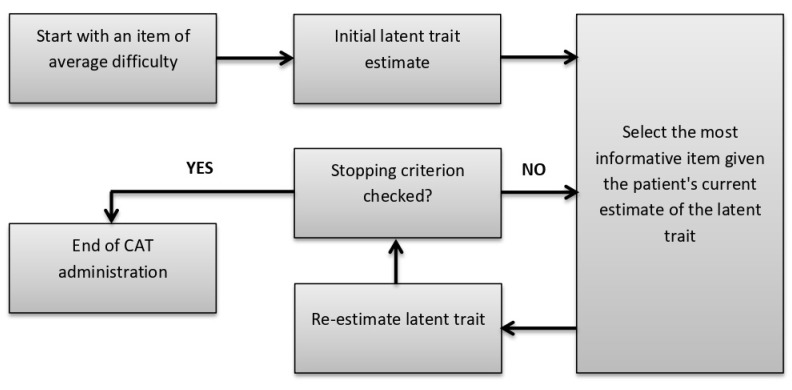
CAT algorithm.

The CAT algorithm began by selecting the starting item based on the maximum Fisher information (MFI) criterion [52]. Based on the response to this item, an initial latent trait estimate (θ) was calculated using the EAP estimate [51]. The CAT algorithm then selected as the next item the item with the highest information for the current θ estimate. The θ estimate was iteratively re-estimated based on the responses to previous items using the EAP estimate. Finally, the CAT algorithm ended when the stopping rule used was reached, which corresponded to the prespecified level of measurement precision based on the standard error of measurement (SEM) [53]. An acceptable range was defined as 0.33 to 0.55, corresponding to reliability coefficients between 0.90 and 0.70 (53). Three scenarios with different stopping rules corresponding to SEM values of 0.33, 0.44, and 0.55 were simulated and compared using the following accuracy and precision indicators: correlation coefficients (r) between CAT scores and scores based on the full set of items in the bank with expected values ≥ 0.90 and the root mean square error (RMSE) with expected values ≤ 0.30 [54]. Figure 1 presents the CAT algorithm.

All of the statistical analyses were performed using IBM PASW SPSS version 20.0 [55], MPlus version 7.0 [56], and R version 4.0.5 [57], with the packages “mirt” [58], “lordif” [59], “BifactorIndicesCalculator” [60], and “mirtCAT” [61].

## 3. Results

### 3.1. Characteristics of the Sample

Of the 541 SMIs patients participating in this study, 93.2% were outpatients and 6.8% were inpatients (43.2% of whom were involuntarily committed). The majority of patients had a diagnosis of schizophrenia (61.9%), and to a lesser extent a diagnosis of bipolar disorders (19.6%) or major depressive disorders (18.5%). Most patients were men (54.7%), single (75.1%), with an education level of bachelor’s degree or higher (76.9%), and unemployed (72.1%). The median age was 36.0 years (28.0–45.0) and the median duration of illness was 6.0 years (2.7–18.0). The characteristics of the sample are provided in Table 1.

### 3.2. Descriptive Analysis

For the initial 37-item pool, the mean ranged from 1.42 ± 1.29 to 3.17 ± 0.86. The floor and ceiling effects ranged from 1.5 to 27.9% and from 6.8 to 35.5%, respectively. Each item had an acceptable skewness coefficient (ranging from −1.45 to 2.04), and missing values ranged from 0.4% to 49.7%. Inter-item correlation coefficients ranged from 0.03 to 0.82. Following this step, seven items (i.e., DT3, DT6, DT11, DT16, DT20, DT21, and DT30) were removed because they exhibited high inter-item correlations (>0.72). These findings are reported in Table 2.

### 3.3. Evaluation of the Assumptions of an IRT Model

The fit indices of the one-factor CFA model were not adequate (RMSEA = 0.152, 95% CI [0.147–0.157], CFI = 0.761, and TLI = 0.743). In the EFA, four factors had eigenvalues greater than 1 (11.4, 3.3, 2.3, and 1.1, respectively). The ratio between the first and second eigenvalues was 3.5, and the total amount of variance explained by the first factor was 38.0%. The scree plot revealed three predominant factors, whereas parallel analysis revealed four predominant factors. Among the 26 items in the bank, 20 items were recoded after examining the item characteristic curves (ICCs). The deviations (final model–initial model) of the AIC and BIC were −9611.67 and −9783.41, respectively, indicating an overall improvement in model fit. Next, we tested a bifactor structure with a general factor and three group factors, which showed adequate fit indices (RMSEA = 0.069, 95% CI [0.062–0.076], CFI = 0.969, TLI = 0.963) after excluding four items (i.e., DT27, DT28, DT34, and DT37) which did not have sufficient factor loading on the first factor (>0.40). The ωh coefficient for the general factor was 0.901, and those of the three group factors were 0.002, 0.431, and 0.697, respectively. The percentage of ECV attributable to the general factor was 69.3%, whereas the remaining 30.7% was attributable to the three group factors (5.4%, 11.7% and 13.7%, respectively). Moreover, Cronbach’s alpha was 0.91 and no residual correlation was above 0.25. We can therefore consider that the 26 items of the PREMIUM-DT bank capture a sufficiently unidimensional construct to perform IRT analyses.

### 3.4. Calibration and Fitting of an IRT Model to the Data

The fit indices of the PCM were less adequate than those of the GPCM (24,490.44 and 23,824.60 for the AIC and 24,769.51 and 24,211.00 for the BIC, respectively), and the likelihood ratio test indicated a better fit of the GPCM compared with the PCM, X^2^= 715.84, *p* < 0.001. As such, we decided to use the GPCM to calibrate the PREMIUM-DT item bank. All items showed an adequate fit to the GPCM with respect to infit values ranging from 0.78 (item DT17) to 1.15 (item DT19). The discrimination parameters ranged from 0.48 (item DT35) to 2.70 (item DT8), and the threshold parameters ranged from −1.96 (item DT12) to 3.25 (item DT35) (Appendix A). 

**Figure 2:**
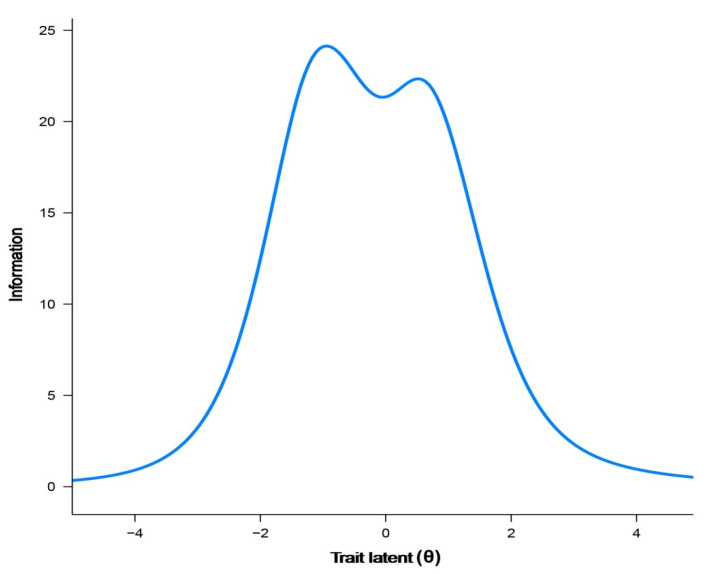
Test information curve of the final PREMIUM-DT item bank.

As shown in Figure 2, the 26 items in the final PREMIUM-DT item bank showed high measurement precision over a wide range of the latent trait (77.3% of total information is included in the [−2, 2] range of the latent continuum values). Item 17 (“You got answers to all your questions about your drug therapy”) was the most informative of the bank, whereas item 35 (“The drug therapy has interfered with your sexuality”) was the least informative.

### 3.5. Evaluation of Differential Item Functioning (DIF)

Of the 104 tests performed (26 items with 4 confounding factors), 10 had overall DIF. According to Zumbo’s classification, no items were flagged for moderate or large DIF magnitudes, and only some items were flagged for negligible DIF magnitudes, and thus were kept in the item bank (Appendix B): three items for sex (items 14, 15, and 36), two items for age (items 10 and 35), three items for care setting (items 8, 9, and 25), and four items for diagnosis (items 9, 15, 23, and 29). 

### 3.6. External Validity of the Item Bank

The mean PREMIUM-DT score was 45.97 ± 16.88. The scores were significantly higher for women, employed individuals, patients with illness duration < 5 years, and those who reported good medication adherence. Age was not correlated with PREMIUM-DT score and no significant difference was found for education level, marital status, care setting and global functioning. PREMIUM-DT scores were significantly different according to diagnosis, and patients with schizophrenia had lower scores than those with bipolar and major depressive disorders. The scores were weakly correlated with scores on SF-12 dimensions measuring physical functioning (PF), social functioning (SF), role physical (RP), role emotional (RE), mental health (MH), vitality (VT), general health (GH) and bodily pain (BP), and both physical quality of life (PCS) and mental quality of life (MCS) composite scores. Finally, PREMIUM-DT scores were highly correlated with scores on the item and VAS of satisfaction with drug therapy. All the details are reported in Table 3.

### 3.7. Elaboration of Item Administration Algorithm

Between the three scenarios, the CAT simulation with SEM < 0.33 was the highest performing, with the highest levels of accuracy (r = 0.97) and precision (RMSE = 0.25) while administering less than half of the items of the PREMIUM-DT item bank (on average eight items). The two other simulations were not satisfactory despite a smaller average number of items administered (four and two items, respectively), with lower-than-expected levels of precision and/or accuracy (for SEM < 0.55, RMSE = 0.46, and r = 0.88, and for SEM < 0.44, RMSE = 0.35, and r = 0.93). Table 4 shows the results of the CAT simulation.

## 4. Discussion

The final PREMIUM-DT item bank of drug therapy experience for adult patients with SMIs demonstrated good psychometric properties (Appendix C). The 26 items showed suitable unidimensionality, evidence of construct validity, and were exempt from moderate- to large-magnitude DIFs regarding sex, age, care setting, and diagnosis. The threshold parameters reflected the overall latent continuum and all items had adequate discriminatory power (i.e., α > 0.50), except for item DT35 (α = 0.48), but it was kept due to its content relevance. The precision of the PREMIUM-DT item bank was highest for θ scores between −2 and 2 and thus covers a broad spectrum of drug therapy experiences. The PREMIUM-DT item bank could be used to facilitate dialogue between clinicians and patients and improve understanding of factors that affect treatment outcomes and patients’ care experiences. This item bank was designed to be administered as a CAT and provides the basis for the development of static brief forms, which allow more accurate and flexible assessments for a wide range of treatments across diverse outpatient and inpatient settings for adult patients with SMIs. 

External validity explored with well-established measures and clinical and sociodemographic data has globally supported our assumptions. Women may report better drug therapy experience due to psychological factors (such as higher commitment to follow-up and treatment [62] and better observance and adherence [63]). Conflicting results were found for the association of gender with weight gain under antipsychotics [64,65], and further studies should explore the role of gender in drug therapy experience [66]. Patients with schizophrenia reported lower drug therapy experience scores than those with bipolar disorder and major depressive disorders. Schizophrenia is known to have a poorer prognosis than bipolar disorders, and major depressive disorders and antipsychotics induce more severe side effects than antidepressants and mood stabilizers [67]. Schizophrenia may also induce paranoid delusions and cognitive impairment that may alter drug therapy experience. Our results also suggest that drug therapy experience is lower at the beginning of the illness, probably due to patient factors (e.g., lower acceptance of the illness) and biological factors (higher side effects at first administration). Individuals who reported better adherence to treatment, as measured with the MARS scale, also reported a better drug therapy experience, which is consistent with other research that has shown that poor adherence is associated with increased subjective side effects [19,68]. Finally, consistent with our hypotheses, the association between drug therapy experience and quality of life, as measured by the SF-12 scale, was positive but weak, which is consistent with both these measures providing two different and complementary perspectives [69,70]. 

The final PREMIUM-DT item bank explores topics of importance to patients that should be considered by clinicians, such as treatment options (DT1), shared decision-making (DT2, DT4, DT5, DT7, and DT8), explanations about practical treatment modalities (DT9, DT10, DT12, DT13, DT14, DT15, and DT17), treatment convenience (DT18), subjective positive effects (DT19, DT22, DT23, DT24, DT25, and DT26) and subjective negative side effects (DT29, DT31, DT32, DT33, DT35, and DT36). In particular, the lowest scores were obtained on items assessing subjective treatment side effects, such as energy, alertness, cognitive impairment and sexuality (i.e., DT29, DT33, and DT35). Respondents also reported poorer experience with practical information, such as what to do if they forget to take their treatment or in case of an excessive dose (i.e., DT15). These findings suggest that practical information and information about the side effects of psychotropic treatments should be targeted as a priority to improve patients’ experience of drug therapy. Finally, the PREMIUM-DT-CAT only needs an average of 8 items to achieve a level of precision based on SEM < 0.33 (corresponding to a reliability >0.90), allowing for a brief and accurate assessment of drug therapy experience for adult patients with SMIs.

### Limitations

First, although our sample size may be considered limited compared to other similar initiatives, it was large enough to calibrate the item pool [71,72]. Patients were recruited from one French university hospital (Assistance Publique—Hôpitaux de Marseille). Nevertheless, other patients were also recruited from expert centers of the FondaMental Foundation and through an online survey on the Internet. Therefore, our sample included both inpatients and outpatients from different facilities. Second, we used the GPCM in this study similarly to another calibration of the PREMIUM item bank [40]. Third, GAF scores and SF-12 dimension scores were reported for a subsample of participants. However, the associations were consistent with our underlying assumptions in the external validity testing. Fourth, the precision and accuracy of the PREMIUM-DT-CAT were adequate but should be re-evaluated in an independent sample than the one used for the IRT model calibration. Fifth, since the aim of this study was to calibrate an item bank measuring patients’ reported experiences with their drug therapy as a whole, no information regarding treatment types was collected. This will be further explored in real-life applications using a digital platform, which is the next step in the PREMIUM project. Finally, external validity evidence was obtained in a sample with a mean duration of illness of 10 years. Further studies should explore the experience of drug therapy at the beginning of psychiatric care. The response profile of psychiatric patients would probably be different than those with an older follow-up. Other unreported data may have influenced the patient’s experience of drug therapy, such as the age, seniority and gender of the therapist and the type of psychotropic treatments. Future work addressing the acceptability by stakeholders and the validity of the PREMIUM-DT-CAT will need to be conducted to explore the generalizability of these findings. 

## 5. Conclusions

The PREMIUM-DT item bank demonstrated strong psychometric properties, and the associated CAT has attractive features for widespread use in clinical settings by offering a briefer and more accurate assessment of the experience of drug therapy for adult patients with SMIs than with standard questionnaires. To conclude, our results suggest that information about the side effects of psychotropic treatment and how to cope with them should be targeted as a priority to improve patients’ experience of drug therapy.

## Figures and Tables

**Table 1:** Sample characteristics (n = 541).

	n(%) Mean ± Standard Deviation Median (Interquartile Range)
**Sociodemographic data**	
Sex (men) (n = 541)	296 (54.7)
Age, years (n = 539)	36.0 (28.0–45.0)
Marital status (single) (n = 510)	383 (75.1)
Educational level (<bachelor’s degree) (n = 511)	118 (23.1)
Employment status (unemployed) (n = 451)	325 (72.1)
**Clinical data**	
Care setting (n = 541)	
Outpatient	504 (93.2)
Inpatient	37 (6.8)
Involuntary commitment	16 (43.2)
Diagnosis (n = 541)	
Schizophrenia	335 (61.9)
Bipolar disorders	106 (19.6)
Major depressive disorders	100 (18.5)
Duration of illness, years (n = 482)	6.0 (2.7–18.0)
<5 years	214 (44.4)
≥5 years	268 (55.6)
Global functioning (GAF score) (n = 327)	55.0 (45.0–68.0)
Poor functioning (<61)	161 (65.7)
Good functioning (≥61)	84 (34.3)
Medication adherence (MARS score) (n = 524)	6.0 (4.0–8.0)
Poor adherence (<7)	305 (58.2)
Good adherence (≥7)	219 (41.8)
Quality of life (SF-12 score)	
PF (n = 396)	46.53 ± 10.47
SF (n = 370)	34.38 ± 10.57
RP (n = 369)	39.69 ± 9.75
RE (n = 370)	32.46 ± 10.96
MH (n = 370)	45.10 ± 9.60
VT (n = 370)	50.77 ± 9.82
BP (n = 370)	43.53 ± 12.88
GH (n = 370)	34.31 ± 9.67
PCS (n = 368)	43.47 ± 9.77
MCS (n = 369)	39.01 ± 9.90

**Abbreviations**: GAF—global assessment of functioning; MARS—medication adherence report scale; SF-12—medical outcome study 12-item Short Form; PF—physical functioning; SF—social functioning; RP—role physical; RE—role emotional; MH—mental health; VT—vitality; BP—bodily pain; GH—general health; PCS—physical composite quality of life score; MCS—mental composite quality of life score.

**Table 2:** Descriptive analysis of the PREMIUM-DT item bank.

Item No.	Content Item	Floor Effect (%)	Ceiling Effect (%)	Missing Values (%)	Skewness Coefficient
**DT1**	The different options (available drug therapies, etc.) have been explained to you	7.4	20.1	4.1	−0.56
**DT2**	Your opinion about the drug therapy has been taken into account	5.0	26.2	2.2	−0.77
**DT3**	You felt comfortable to discuss the drug therapy	4.8	29.8	1.7	−0.90
**DT4**	You have been involved in decisions (choice of drug therapy, its modifications, etc.)	5.4	22.9	1.5	−0.57
**DT5**	You knew who to contact to ask questions about your drug therapy	5.0	28.8	2.6	−1.05
**DT6**	You knew who to contact to change or adjust your drug therapy	5.2	29.2	4.4	−1.08
**DT7**	Your medical history (allergies, previous treatments, previous and current illnesses...) have been taken into account in the choice of your drug therapy	4.8	27.4	8.5	−1.06
**DT8**	The interest of the drug therapy has been explained to you	3.9	28.8	2.8	−1.06
**DT9**	The timeframe for the drug therapy to become effective has been explained to you	5.2	21.3	5.2	−0.76
**DT10**	The total duration of the drug therapy (end date or long-term continuation) has been explained to you	10.4	20.0	3.5	−0.24
**DT11**	How to deal with side effects has been explained to you	13.5	12.6	4.1	0.08
**DT12**	How to take your medication has been explained to you (when, how many times a day/month, etc.)	1.8	35.5	3.5	−1.45
**DT13**	The side effects of the drug therapy have been explained to you	13.7	13.3	2.8	−0.06
**DT14**	The expected consequences of your drug therapy on your health (physical, mental or in your social life, especially with your relatives) have been explained to you	11.3	15.3	2.8	−0.27
**DT15**	Practical instructions have been given to you (what to do if you forget, in case of excessive dose, etc.)	16.3	11.8	5.5	0.07
**DT16**	You think you have received all the important information about your drug therapy	10.4	16.6	2.0	−0.38
**DT17**	You received answers to all your questions about your drug therapy	8.9	18.5	3.0	−0.46
**DT18**	The frequency and schedules of your drug therapy was convenient for you	2.4	29.6	1.8	−1.07
**DT19**	The drug therapy has helped you in your daily life	4.8	29.2	0.9	−1.02
**DT20**	The drug therapy has improved your well-being	7.2	25.9	0.7	−0.84
**DT21**	The drug therapy has met your needs	6.8	19.4	0.7	−0.63
**DT22**	The drug therapy has been effective for your health problem	1.5	12.2	48.4	−0.90
**DT23**	The drug therapy has helped you solve your problems	9.2	14.4	1.1	−0.34
**DT24**	The drug therapy has helped you feel more confident in yourself	13.7	10.7	1.8	−0.12
**DT25**	The drug therapy has been tailored to your health status	5.0	22.9	0.6	−0.81
**DT26**	You were confident in the interest and effectiveness of your drug therapy	6.5	22.7	0.4	−0.78
**DT27**	The side effects were bothersome to you *	27.9	8.5	4.4	2.04
**DT28**	The drug therapy was accompanied by an embarrassing weight gain *	16.8	6.8	49.7	1.59
**DT29**	The drug therapy has interfered with your energy *	25.7	9.4	1.5	1.65
**DT30**	The drug therapy has interfered with your motivation *	18.7	12.2	1.7	1.30
**DT31**	The drug therapy has interfered with the quality of your sleep *	11.8	17.2	1.8	1.08
**DT32**	The drug therapy has made you irritable and moody *	5.7	23.5	2.8	1.32
**DT33**	The drug therapy has interfered with your alertness (thinking clearly, staying awake, etc.) *	16.1	13.1	1.7	1.37
**DT34**	The drug therapy has interfered with your memory and concentration *	23.1	9.4	1.7	1.62
**DT35**	The drug therapy has interfered with your sexuality *	21.6	9.8	9.4	1.55
**DT36**	The side effects of your drug therapy have been taken into account	9.2	10.9	6.5	−0.20
**DT37**	You have thought that another drug therapy would have suited you better	8.1	14.4	4.3	1.85

**Notes:** * items negatively worded and reverse scored for subsequent analyses.

**Table 3:** Comparison of PREMIUM-DT scores with sociodemographic and clinical data and proxy measures of quality of care.

	Correlation Coefficient (r)	Mean ± Standard Deviation	*p* Value
**Sociodemographic data**			
Age	0.03	-	0.506
Sex	-		0.022
Men		44.80 ± 15.69	
Women		48.17 ± 17.68	
Marital status	-		0.133
Single		45.35 ± 16.93	
Non-single		47.98 ± 17.32	
Educational level	-		0.766
<Bachelor’s degree		45.61 ± 18.63	
≥Bachelor’s degree		46.14 ± 16.55	
Employment status	-		0.041
Employed		48.75 ± 17.44	
Unemployed		45.05 ± 17.19	
**Clinical data**			
Care setting	-		0.611
Outpatient		45.87 ± 16.98	
Inpatient		47.34 ± 15.64	
Diagnosis	-		0.024
Schizophrenia		44.51 ± 15.79	
Bipolar disorders		49.38 ± 19.00	
Major depressive disorders		47.28 ± 17.59	
Duration of illness	-		0.007
<5 years		48.55 ± 16.21	
≥5 years		44.33 ± 17.52	
Global functioning (GAF score)	-		0.423
Poor functioning (<61)		47.65 ± 15.76	
Good functioning (≥61)		49.31 ± 14.51	
Medication adherence (MARS score)			<0.001
Poor adherence (<7)		48.61 ± 15.62	
Good adherence (≥7)		43.30 ± 17.20	
**Proxy measures**			
Item of overall satisfaction	0.65	-	<0.001
VAS	0.66	-	<0.001
Quality of life (SF-12)			
PF	0.21	-	<0.001
SF	0.27	-	<0.001
RP	0.29	-	<0.001
RE	0.27	-	<0.001
MH	0.25	-	<0.001
VT	0.19	-	<0.001
BP	0.31	-	<0.001
GH	0.26	-	<0.001
PCS	0.28	-	<0.001
MCS	0.24	-	<0.001

**Abbreviations**: GAF—global assessment of functioning; MARS—medication adherence report scale; VAS—visual analogue scale; SF-12—medical outcome study 12-item Short Form; PF—physical functioning; SF—social functioning; RP—role physical; RE—role emotional; MH—mental health; VT—vitality; BP—bodily pain; GH—general health; PCS—physical composite quality of life score; MCS—mental composite quality of life score.

**Table 4:** Mean scores and precision indicators for CAT simulation.

Precision Level	Indicators	
**SEM < 0.33**	Median (IQR)	44.3 (36.6–54.0)
	Correlation coefficient (r)	0.97
	RMSE	0.25
	Mean number of items	7.77
**SEM < 0.44**	Median (IQR)	44.8 (34.8–53.1)
	Correlation coefficient (r)	0.93
	RMSE	0.35
	Mean number of items	3.86
**SEM < 0.55**	Median (IQR)	52.5 (35.4–68.1)
	Correlation coefficient (r)	0.88
	RMSE	0.46
	Mean number of items	1.96

**Abbreviations**: IQR—interquartile range; SEM—standard error of measurement; RMSE—root mean square error.

## Data Availability

The data are available on demand from the PREMIUM Scientific Committee.

## Supplementary Materials

The following supporting information can be downloaded at: https://www.mdpi.com/article/10.3390/jcm11154278/s1, Evaluation of the assumptions of the IRT model.

## Appendix A

**Table A1:** Parameter estimates (discrimination and thresholds) and fit statistics for the 26 items in the final PREMIUM-DT item bank.

**Item No.**	**Discrimination**	**Threshold 1**	**Threshold 2**	**Threshold 3**	**Threshold 4**	**Infit**
**DT1**	2.24	−0.85	0.92	-	-	0.95
**DT2**	2.52	−1.11	0.64	-	-	0.99
**DT4**	1.85	−0.98	0.88	-	-	1.02
**DT5**	2.31	−1.29	0.58	-	-	0.78
**DT7**	2.26	−1.38	0.57	-	-	0.94
**DT8**	2.70	−1.27	0.53	-	-	0.97
**DT9**	2.17	−1.06	0.88	-	-	1.00
**DT10**	1.86	−0.53	0.96	-	-	0.95
**DT12**	1.93	−1.96	0.36	-	-	0.85
**DT13**	1.87	−0.35	1.39	-	-	0.85
**DT14**	2.24	−0.56	1.19	-	-	1.04
**DT15**	1.44	−0.14	1.57	-	-	0.94
**DT17**	1.93	−1.35	−0.62	−0.44	1.01	0.78
**DT18**	2.08	−1.50	0.59	-	-	0.94
**DT19**	1.78	−1.56	0.66	-	-	1.15
**DT22**	1.56	−1.68	1.11	-	-	0.97
**DT23**	0.62	−1.53	−1.34	−0.16	1.83	0.90
**DT24**	0.57	−1.33	−0.55	−0.08	2.33	0.96
**DT25**	1.51	−1.56	−1.34	−0.73	0.91	0.98
**DT26**	1.24	−1.39	−1.26	−0.80	0.97	0.80
**DT29**	0.54	1.18	2.66	-	-	1.06
**DT31**	0.64	−1.07	2.03	-	-	1.07
**DT32**	0.72	−1.90	1.45	-	-	1.06
**DT33**	0.64	0.07	2.19	-	-	1.05
**DT35**	0.48	0.02	3.25	-	-	1.09
**DT36**	0.84	−1.73	−0.48	−0.26	1.89	0.85

## Appendix B

**Table A2:** DIF results.

**Item No.**	**Sex**		**Age**		**Care Setting**		**Diagnosis**	
	***p*** **Value**	**ΔR^2^**	***p*** **Value**	**ΔR^2^**	***p*** **Value**	**ΔR2**	***p*** **Value**	**ΔR^2^**
**DT1**	0.699	-	0.108	-	0.513	-	0.061	-
**DT2**	0.449	-	0.261	-	0.072	-	0.601	-
**DT4**	0.537	-	0.308	-	0.221	-	0.247	-
**DT5**	0.781	-	0.734	-	0.361	-	0.687	-
**DT7**	0.898	-	0.667	-	0.165	-	0.164	-
**DT8**	0.387	-	0.224	-	**0.006**	**0.010**	0.366	-
**DT9**	0.214	-	0.169	-	**0.005**	**0.011**	**0.014**	**0.013**
**DT10**	0.319	-	**0.002**	**0.011**	0.434	-	0.113	-
**DT12**	0.362	-	0.288	-	0.673	-	0.140	-
**DT13**	0.122	-	0.731	-	0.379	-	0.367	-
**DT14**	**0.003**	**0.012**	0.364	-	0.104	-	0.943	-
**DT15**	**0.001**	**0.015**	0.402	-	0.117	-	**0.009**	**0.014**
**DT17**	0.178	-	0.516	-	0.259	-	0.273	-
**DT18**	0.839	-	0.718	-	0.879	-	0.716	-
**DT19**	0.262	-	0.357	-	0.588	-	0.257	-
**DT22**	0.495	-	0.278	-	0.817	-	0.229	-
**DT23**	0.057	-	0.080	-	0.825	-	**0.000**	**0.021**
**DT24**	0.503	-	0.231	-	0.281	-	0.034	-
**DT25**	0.764	-	0.491	-	**0.005**	**0.008**	0.320	-
**DT26**	0.070	-	0.978	-	0.130	-	0.523	-
**DT29**	0.780	-	0.067	-	0.013	-	**0.001**	**0.020**
**DT31**	0.678	-	0.187	-	0.621	-	0.765	-
**DT32**	0.620	-	0.103	-	0.611	-	0.134	-
**DT33**	0.952	-	0.816	-	0.960	-	0.265	-
**DT35**	0.903	-	**0.008**	**0.010**	0.551	-	0.044	-
**DT36**	**0.008**	**0.006**	0.213	-	0.703	-	0.135	-

**Notes:** Bold values indicate DIF *p* value < 0.01. ΔR^2^: DIF magnitude: negligible (ΔR^2^ < 0.13), moderate (0.13 ≤ ΔR^2^ ≥ 0.26), or large (ΔR^2^ ≥ 0.26).

## Appendix C

**Table A3:** List of the 26 items of the PREMIUM-DT item bank (English and French versions).

**Item No.**	**Item Content in English**	**Item Content in French**
**DT1**	The different options (available drug therapies, etc.) have been explained to you	Les différentes options (les traitements médicamenteux disponibles, etc.) vous ont été expliquées
**DT2**	Your opinion about the drug therapy has been taken into account	Votre avis a été pris en compte sur le traitement médicamenteux
**DT4**	You have been involved in decisions (choice of drug therapy, its modifications, etc.)	Vous avez été impliqué(e) dans les décisions (le choix du traitement médicamenteux, ses modifications, etc.)
**DT5**	You knew who to contact to ask questions about your drug therapy	Vous avez su à qui vous adresser pour poser des questions sur votre traitement médicamenteux
**DT7**	Your medical history (allergies, previous treatments, previous and current illnesses...) have been taken into account in the choice of your drug therapy	Vos antécédents médicaux (allergies, traitements précédents, maladies passées et actuelles…) ont été pris en compte dans le choix de votre traitement médicamenteux
**DT8**	The interest of the drug therapy has been explained to you	L’intérêt du traitement médicamenteux vous a été expliqué
**DT9**	The timeframe for the drug therapy to become effective has been explained to you	Le délai pour que le traitement médicamenteux devienne efficace vous a été expliqué
**DT10**	The total duration of the drug therapy (end date or long-term continuation) has been explained to you	La durée totale du traitement médicamenteux (date d’arrêt ou poursuite au long cours) vous a été expliquée
**DT12**	How to take your medication has been explained to you (when, how many times a day/month, etc.)	Les modalités de la prise médicamenteuse vous ont été expliquées (à quel moment, combien de fois par jour/mois, etc.)
**DT13**	The side effects of the drug therapy have been explained to you	Les effets secondaires du traitement médicamenteux vous ont été expliqués
**DT14**	The expected consequences of your drug therapy on your health (physical, mental or in your social life, especially with your relatives) have been explained to you	Les conséquences attendues de votre traitement médicamenteux sur votre santé (physique, mentale ou dans votre vie sociale notamment avec votre entourage) vous ont été expliquées
**DT15**	Practical instructions have been given to you (what to do if you forget, in case of excessive dose, etc.)	Des consignes pratiques vous ont été données (que faire en cas d’oubli, de surdosage, etc.)
**DT17**	You received answers to all your questions about your drug therapy	Vous avez obtenu des réponses à toutes vos questions sur votre traitement médicamenteux
**DT18**	The frequency and schedules of your drug therapy was convenient for you	La fréquence et les horaires auxquels vous avez dû prendre votre traitement médicamenteux vous ont convenu
**DT19**	The drug therapy has helped you in your daily life	Le traitement médicamenteux vous a aidé dans votre vie de tous les jours
**DT22**	The drug therapy has been effective for your health problem	Le traitement médicamenteux a été efficace sur votre problème de santé
**DT23**	The drug therapy has helped you solve your problems	Le traitement médicamenteux vous a aidé(e) à résoudre vos problèmes
**DT24**	The drug therapy has helped you feel more confident in yourself	Le traitement médicamenteux vous a aidé(e) à avoir davantage confiance en vous
**DT25**	The drug therapy has been tailored to your health status	Le traitement médicamenteux a été adapté à votre état de santé
**DT26**	You were confident in the interest and effectiveness of your drug therapy	Vous avez eu confiance dans l’intérêt et l’efficacité de votre traitement médicamenteux
**DT29**	The drug therapy has interfered with your energy	Le traitement médicamenteux a modifié de façon gênante votre énergie
**DT31**	The drug therapy has interfered with the quality of your sleep	Le traitement médicamenteux a modifié de façon gênante la qualité de votre sommeil
**DT32**	The drug therapy has made you irritable and moody	Le traitement médicamenteux vous a rendu irritable et susceptible
**DT33**	The drug therapy has interfered with your alertness (thinking clearly, staying awake, etc.)	Le traitement médicamenteux a modifié de façon gênante votre vigilance (avoir les idées claires, rester éveillé, etc.)
**DT35**	The drug therapy has interfered with your sexuality	Le traitement médicamenteux a modifié de façon gênante votre sexualité
**DT36**	The side effects of your drug therapy have been taken into account	Les effets secondaires ont été pris en compte
