# Supplementary material for: Calibration and Validation of a PREMIUM-DT Item Bank to Measure the Experience of Drug Therapy for Patients with Severe Mental Illness
Source: J Clin Med. 2022 Jul 22;11(15):4278. doi: 10.3390/jcm11154278 (PMC9331305; doi:10.3390/jcm11154278)
Supplement: Supplementary file 1 [file jcm-11-04278-s001.zip › jcm-1745793-supplementary.pdf]

Supplementary materials.

Evaluation of the assumptions of the IRT model.

The unidimensionality assumption was evaluated based on a 1-factor confirmatory factor analysis (CFA) with the weighted least square mean and variance (WLSMV) estimator due to the ordinal nature of the data [29]. The following indices were used to assess the goodness-of-fit of the model to the data, with an acceptable fit defined by: the root mean square error of approximation (RMSEA) ≤ 0.08, the comparative fit index (CFI), and the Tucker–Lewis index (TLI) ≥ 0.95 [30,31]. If the CFA showed poor fit, an exploratory factor analysis (EFA) was performed after randomly dividing the entire sample into two subsamples (n=270 for EFA and n=271 for CFA). The number of factors to be kept was based on several criteria [27]: Kaiser-Guttman's rule (eigenvalues ≥1), differences in the magnitude of eigenvalues between factors (a ratio greater than 4 is expected), the scree test (looking for an "elbow" in the curve), parallel analysis and factor loadings (with minimum item loadings set at 0.40). Next, to investigate whether item responses are sufficiently unidimensional for IRT application, we used a bifactor model [32]. The bifactor model assumes one general factor (in this case, the patients' experience of drug therapy), onto which all items load, and several group factors, onto which unique subsets of items load (32). The percentage of explained common variance (ECV) and the omega hierarchical (ω_h/ω_{hs}) coefficients accounted for by the general factor and by group factors were calculated, with an expected ω_h coefficient for the general factor greater than or equal to 0.70 and the expected percentage of ECV for the general factor greater than or equal to 60% to support unidimensionality [33,34].

Local independence was examined using residual correlations from the final CFA model. All residual correlations greater than 0.20 (or 0.25) indicated possible local dependence, leading to the deletion of the item with the highest residual correlation with other items in the bank [35,36].

Finally, monotonicity was evaluated by visual inspection of item characteristic curves (ICCs), with each response category expected to have a maximum probability of being selected on a specific range of the latent trait continuum. If two categories were not sufficiently discriminative for a particular item, they were collapsed, and the resulting model was re-estimated. The deviations of the Akaike information criterion (AIC)[37] and the Bayes information criterion (BIC)[38] between the final model (recoded items) and the initial model (no recoded items) were computed to ensure that the recoding process resulted in a substantial improvement in the model.
